# Real-World Concordance of Indirect Immunofluorescence and Antigen-Specific Assays in Antineutrophil Cytoplasmic Antibody Testing: A Retrospective High-Volume Laboratory Study

**DOI:** 10.5152/eurasianjmed.2026.261498

**Published:** 2026-08-10

**Authors:** Ayşe Arslan, Alper Togay

**Affiliations:** Department of Medical Microbiology, İzmir City Hospital, İzmir, Türkiye

**Keywords:** Antineutrophil cytoplasmic antibodies, indirect immunofluorescence, laboratory diagnostics, myeloperoxidase, proteinase 3

## Abstract

**Background:**

Current guidelines recommend myeloperoxidase (MPO)- and proteinase 3 (PR3)-based immunoassays as the primary approach for antineutrophil cytoplasmic antibody (ANCA) testing, while indirect immunofluorescence (IIF) is reserved for selected clinical contexts. However, real-world data regarding concordance and workflow implications of these approaches remain limited.

**Methods:**

In this retrospective laboratory-based study, ANCA results from 8976 consecutive patients evaluated in a high-volume tertiary laboratory were analyzed. Indirect immunofluorescence patterns and MPO/PR3 reactivity were assessed simultaneously using an integrated IIF-based platform. Concordance between IIF patterns and antigen-specific reactivity was evaluated, and retrospective workflow simulations were performed to estimate the effects of MPO/PR3-first and IIF-first testing approaches on laboratory test utilization.

**Results:**

Overall IIF positivity was observed in 589 patients (6.56%), whereas MPO and/or PR3 reactivity was detected in 307 patients (3.42%; 95% CI: 3.06-3.82%). Among IIF-positive patients, 438 (74.4%) showed no detectable MPO or PR3 reactivity, while 156 antigen-reactive patients (50.8%; 95% CI: 45.1-56.5%) showed no identifiable IIF reactivity. Retrospective workflow simulations suggested that an MPO/PR3-first approach would substantially reduce IIF testing volume, whereas an IIF-first strategy would markedly decrease antigen-specific testing volume. However, each approach was associated with distinct discordance patterns between IIF and MPO/PR3 reactivity within the study cohort.

**Conclusion:**

Substantial discordance was observed between IIF patterns and MPO/PR3 reactivity. The findings reflect laboratory concordance and workflow characteristics rather than true clinical diagnostic performance, and the clinical significance of discordant findings requires further evaluation in clinically characterized cohorts.

**Main Points**

- In this large real-world laboratory cohort, indirect immunofluorescence (IIF) patterns and myeloperoxidase/proteinase 3 (MPO/PR3) reactivity demonstrated limited concordance in routine antineutrophil cytoplasmic antibody (ANCA) testing.
- A substantial proportion of antigen-reactive samples showed no detectable IIF reactivity, while many IIF-positive samples lacked MPO/PR3 reactivity.
- Retrospective workflow simulations suggested that MPO/PR3-first approaches would markedly reduce IIF testing volume, whereas IIF-first approaches would substantially reduce antigen-specific testing volume.
- Different testing approaches were associated with distinct discordance patterns between IIF and MPO/PR3 reactivity within the study cohort.
- These findings reflect laboratory concordance and workflow characteristics rather than clinical diagnostic performance and highlight the complementary but nonidentical characteristics of IIF and antigen-specific assays in routine ANCA testing.

## Introduction

Antineutrophil cytoplasmic antibodies (ANCA) are important serological markers in the diagnostic evaluation of ANCA-associated vasculitides (AAV), a group of immune-mediated small-vessel vasculitides that includes granulomatosis with polyangiitis and microscopic polyangiitis.^1^ Laboratory detection of ANCA constitutes a major component of the diagnostic work-up in patients with suspected AAV.

Historically, ANCA screening has relied predominantly on indirect immunofluorescence (IIF) using ethanol-fixed neutrophils. This method enables visualization of characteristic staining patterns, most notably cytoplasmic (C-ANCA) and perinuclear (P-ANCA) reactivity. The IIF-based strategy was formally endorsed as the recommended initial screening approach in the 1999 international consensus statement on ANCA testing and reporting, which established the foundational framework for laboratory evaluation of AAV for nearly 2 decades.^2^

Subsequent advances in solid-phase immunoassays enabled antigen-specific detection of antibodies directed against proteinase 3 (PR3) and myeloperoxidase (MPO), resulting in improved analytical specificity and greater standardization of ANCA testing across laboratories. In response to these developments, the revised 2017 international consensus recommendations proposed a paradigm shift toward MPO/PR3-based assays as the preferred first-line testing strategy, with IIF repositioned as a complementary or confirmatory method rather than a mandatory initial screening test.^3^ This updated approach aimed to reduce inter-observer variability and improve standardization and efficiency within laboratory workflows.

Despite these recommendations, the implementation of MPO/PR3-first algorithms in routine clinical practice remains heterogeneous. In high-volume tertiary care and referral centers, testing strategies are influenced not only by guideline recommendations but also by practical considerations such as workload, financial constraints, available instrumentation, and clinician ordering patterns.^4^^,^^5^ Consequently, IIF-based ANCA testing continues to be widely used, either as a primary screening tool or in parallel with antigen-specific assays.

Indirect immunofluorescence may still provide additional pattern-level interpretative information through pattern recognition and identification of atypical ANCA reactivity in selected clinical scenarios beyond antigen specificity alone.^5^^,^^6^ From a laboratory stewardship perspective, the complete abandonment of IIF without considering real-world operational constraints may not be feasible in all settings. However, data evaluating the concordance and workflow characteristics of IIF-based ANCA testing in contemporary routine practice—particularly when MPO and PR3 reactivity are simultaneously available—remain limited, especially in large, unselected patient cohorts.

Against this background, the present study aimed to evaluate concordance patterns between IIF-based ANCA testing and MPO/PR3 reactivity in a high-volume tertiary care laboratory using large-scale routine laboratory data. In addition, retrospective workflow simulations were performed to estimate how different testing approaches may influence laboratory test utilization under real-world practice conditions.

## Material and Methods

This retrospective observational study included patients whose serum samples were submitted to the Medical Microbiology Laboratory of İzmir City Hospital for ANCA testing between January 2023 and September 2025. A total of 8976 consecutive patients were evaluated during this period. For individuals with multiple ANCA test requests, only the first available result was included to avoid duplication.

Demographic data and laboratory findings were retrieved from the institutional laboratory information system. The study was conducted in accordance with the principles of the Declaration of Helsinki. Ethical approval was obtained from the Non-Interventional Clinical Research Ethics Committee of İzmir City Hospital (Approval No. 2026/105 dated February 18, 2026). Informed consent was waived due to the retrospective design of the study. This study used ChatGPT (OpenAI, GPT-5.3, 2026) to improve language clarity and edit grammar during the preparation of the manuscript. However, all content of the study was critically reviewed and approved by the authors, who take full responsibility for its accuracy and integrity.

Because the present analysis was based on routine laboratory data retrieved from the laboratory information system, detailed clinical diagnoses, including confirmed ANCA-associated vasculitis, were not systematically available for all patients. The study therefore focuses on laboratory concordance and testing characteristics rather than clinical disease prevalence or diagnostic performance.

Antineutrophil cytoplasmic antibodies testing and antigen-specific antibody detection were performed using a commercially available integrated IIF assay system based on ethanol-fixed neutrophils (Euroimmun AG, Lübeck, Germany). Formalin-fixed neutrophils were evaluated in parallel for pattern confirmation in accordance with the manufacturer’s recommendations. As atypical ANCA patterns were not systematically recorded as a separate category in the laboratory information system, they were not included in the primary analysis.

Serum samples were screened at an initial dilution of 1 : 10. Slides were examined using an immunofluorescence microscope, and fluorescence patterns were evaluated by experienced laboratory personnel. Antineutrophil cytoplasmic antibodies reactivity was classified as C-ANCA, P-ANCA, or negative, according to established fluorescence pattern criteria.

Within the same assay system, antigen-specific antibodies against MPO and PR3 were evaluated using antigen-coated substrates integrated into the assay slide, allowing simultaneous assessment of fluorescence patterns and antigen specificity for each sample.

### Statistical Analysis

Antineutrophil cytoplasmic antibodies positivity rates, distribution of IIF patterns, and MPO/PR3 antibody reactivity were analyzed descriptively. Concordance between IIF patterns and antigen-specific antibody results was evaluated using Cohen’s kappa (κ) coefficient; values were interpreted as follows: κ < 0.20, low; 0.21-0.40, fair; 0.41-0.60, moderate; 0.61-0.80, good; and >0.80, very good agreement. Proportions are reported with 95% CIs where applicable.

Retrospective simulations were performed to model alternative testing algorithms—MPO/PR3-first and IIF-first strategies—to estimate their potential impact on laboratory test utilization and discordance patterns under routine laboratory conditions.

All analyses were performed using IBM SPSS Statistics version 26.0 (IBM Corp., Armonk, NY, USA). The level of statistical significance was set at *P* < .05.

## Results

A total of 8976 unique patients who underwent ANCA testing were included. The study population comprised adult patients referred from multiple clinical departments, reflecting routine diagnostic practice across a broad clinical spectrum. The median age was 51 years (interquartile range = 37-64 years; range, 18-98 years), with a mean of 50.4 years. Overall, 5446 patients (60.7%) were female and 3530 (39.3%) were male.

Test requests were most frequently issued by the departments of Rheumatology (n = 3428; 38.2%), Neurology (n = 915; 10.2%), Internal Medicine (n = 874; 9.7%), Pulmonology (n = 777; 8.7%), Gastroenterology (n = 647; 7.2%), Allergy (n = 604; 6.7%), and Nephrology (n = 443; 4.9%). The distribution of test requests according to the referring clinical departments is summarized in Table 1.

Overall ANCA positivity by IIF was detected in 589 patients (6.56%). Among IIF-positive samples, P-ANCA was observed in 453 patients (5.05% of the total cohort), whereas C-ANCA was detected in 136 patients (1.52%). The remaining 8387 patients (93.44%) were IIF negative.

Myeloperoxidase antibodies were detected in 153 patients (1.70%) and PR3 antibodies in 159 patients (1.77%). Overall, 307 patients (3.42%; 95% CI: 3.06%-3.82%) were positive for MPO and/or PR3 antibodies, including 5 patients (0.06%) with concurrent MPO and PR3 positivity (Table 1).

**Figure 1.**
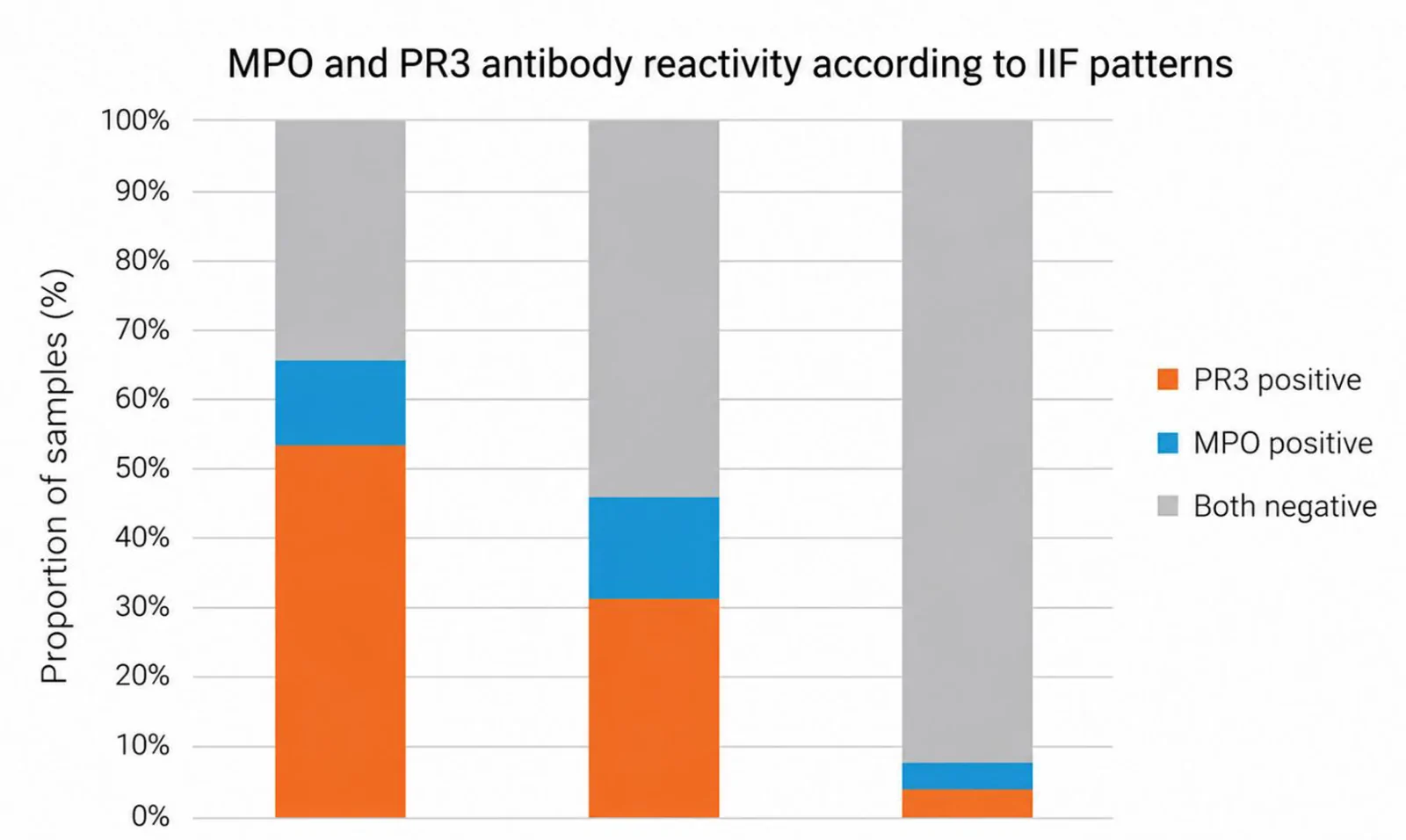
Distribution of MPO and PR3 antibody reactivity according to IIF patterns. Stacked bar chart showing the proportions of PR3-reactive, MPO-reactive, and MPO/PR3-negative samples within each ANCA-IIF pattern category. IIF, indirect immunofluorescence; MPO, myeloperoxidase; PR3, proteinase 3.

Concordance between IIF patterns and antigen-specific ANCA results is summarized in Table 2. Among C-ANCA-positive patients, PR3 antibodies were detected in 66 cases (48.5%) and MPO antibodies in 14 cases (10.3%). Among P-ANCA-positive patients, MPO antibodies were identified in 64 cases (14.1%) and PR3 antibodies in 9 cases (2.0%). Overall, 438 of 589 IIF-positive patients (74.4%) showed no detectable MPO or PR3 reactivity. Conversely, antigen-specific ANCA positivity in the absence of IIF reactivity was observed in 156 patients (50.8%; 95% CI: 45.1-56.5). Overall concordance between IIF patterns and MPO or PR3 antibody detection was fair (κ = 0.306), with moderate agreement for C-ANCA and PR3 positivity (κ = 0.438) and low agreement for P-ANCA and MPO positivity (κ = 0.191; Table 2). The distribution of MPO and PR3 reactivity across IIF patterns is illustrated in Figure 1.

**Figure 2.**
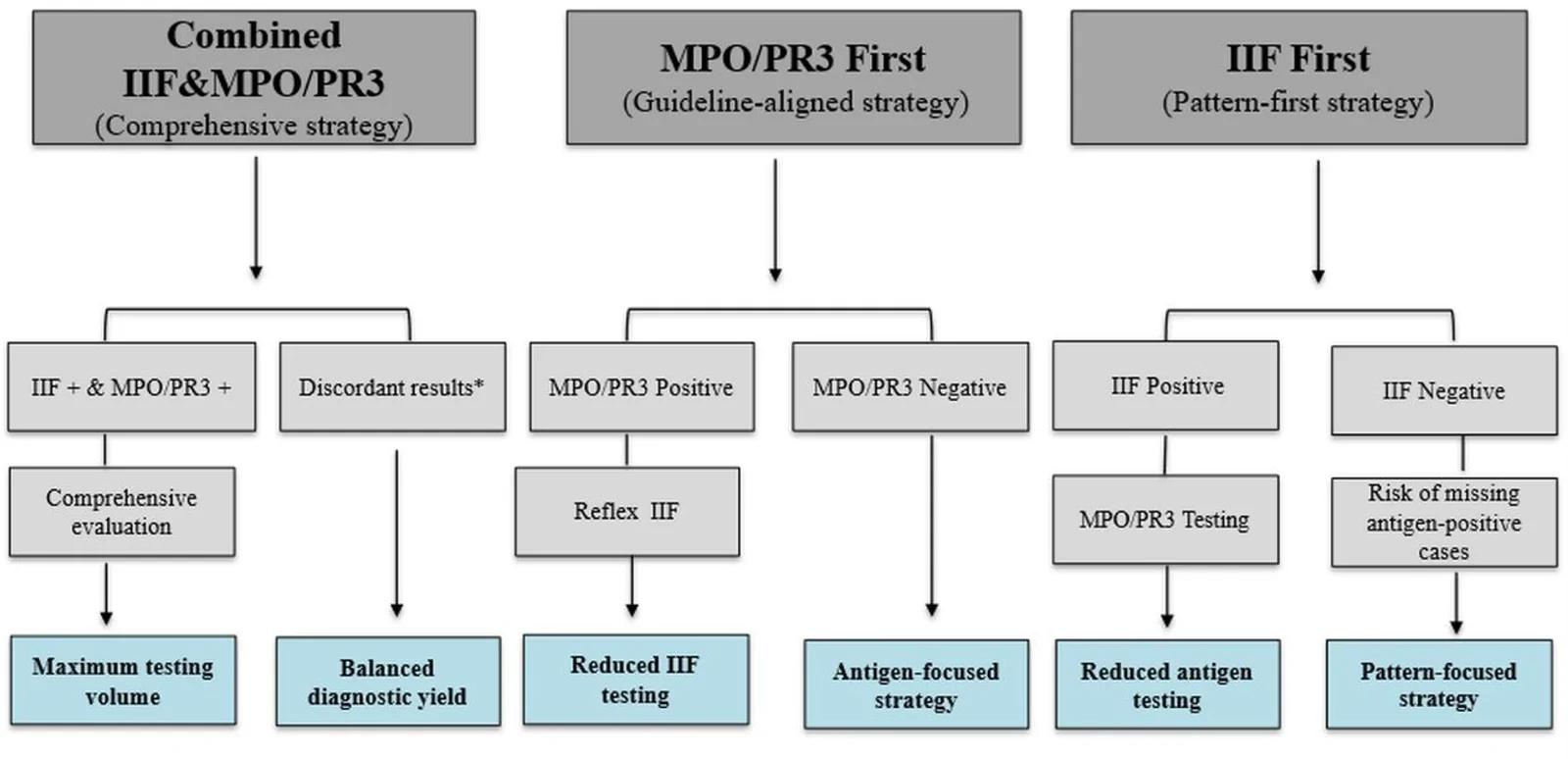
Retrospective workflow simulation of alternative ANCA testing approaches. Schematic representation of 3 diagnostic workflows evaluated in the context of the present study: (1) combined IIF and MPO/PR3 testing performed simultaneously for all samples, (2) MPO/PR3-first testing with reflex IIF in antigen-positive cases, and (3) IIF-first testing with reflex MPO/PR3 analysis in IIF-positive samples. The figure summarizes the relative differences between these approaches in terms of testing volume, workflow characteristics, and potential analytical trade-offs observed in routine laboratory practice. In the present cohort, MPO/PR3-first strategies would substantially reduce IIF testing volume, whereas IIF-first strategies may reduce antigen-specific testing while some MPO/PR3-reactive but IIF-negative samples would not proceed to antigen-specific evaluation during the initial IIF screening step. *Discordant results indicate inconsistency between IIF findings and MPO/PR3 reactivity. ANCA, antineutrophil cytoplasmic antibodies; IIF, indirect immunofluorescence; MPO, myeloperoxidase; PR3, proteinase 3.

Retrospective workflow simulations showed that under a hypothetical MPO/PR3-first approach—with reflex IIF testing restricted to antigen-positive samples—the number of IIF tests would decrease by 96.6%. However, IIF reactivity patterns would not be evaluated in 438 antigen-negative but IIF-positive patients under this model. Conversely, an IIF-first approach—with antigen testing limited to IIF-positive samples—would reduce MPO/PR3 testing by 93.4%, while antigen reactivity without detectable IIF patterns would not be identified within the initial screening step in 156 patients (Figure 2).

## Discussion

In this large-scale, real-world laboratory study involving 8976 consecutive patients, the present study evaluated concordance patterns between IIF-based ANCA testing and MPO/PR3 reactivity under routine laboratory conditions. Overall concordance between IIF patterns and antigen-specific reactivity was limited, with moderate agreement observed between C-ANCA and PR3 reactivity and only low agreement between P-ANCA and MPO reactivity. These findings are consistent with previous studies reporting stronger associations between PR3 antibodies and C-ANCA patterns than between MPO antibodies and P-ANCA patterns.^7^^-^^9^ This asymmetry may reflect both biological differences in antigen–antibody relationships and known technical and interpretative limitations of IIF analysis.^10^^,^^11^ In the present cohort, more than half of antigen-reactive cases showed no concurrent IIF reactivity, while 74.4% of IIF-positive samples lacked MPO or PR3 reactivity, supporting the presence of limited concordance between IIF patterns and MPO/PR3 reactivity in a heterogeneous routine laboratory population.

Although IIF-positive but MPO/PR3-negative cases do not fulfill the serological profile typically associated with classical AAV, reproducible immunofluorescence patterns may still provide additional interpretative information in selected clinical contexts when considered together with clinical and laboratory findings.^12^^-^^14^ However, the clinical significance of isolated IIF reactivity could not be determined from the present dataset because systematic clinical correlation and longitudinal follow-up data were not available. In addition, some discordant IIF-positive findings may reflect the known operator dependency, limited specificity, and interpretative variability of IIF analysis in routine laboratory practice.^10^^,^^11^ Conversely, the substantial proportion of antigen-reactive but IIF-negative cases observed in the present cohort is consistent with the rationale underlying the 2017 international consensus recommendations favoring MPO/PR3-based assays as the primary testing approach.^3^

Retrospective workflow simulations performed within the same dataset suggested that alternative testing approaches may substantially influence laboratory test utilization patterns. An IIF-first approach markedly reduced ­antigen-specific testing volume, whereas antigen-­reactive samples without detectable IIF patterns would not have proceeded to antigen-specific evaluation during the initial screening step.^15^ Conversely, MPO/PR3-first approaches substantially reduced the number of IIF evaluations performed, while IIF reactivity patterns in antigen-negative samples would not have been evaluated within this framework. These findings reflect discordance patterns within the study cohort rather than true clinical diagnostic performance. Accordingly, the clinical implications of isolated IIF positivity or isolated MPO/PR3 reactivity remain uncertain and should be interpreted cautiously in the absence of clinically characterized patient cohorts.^3^^,^^11^^,^^16^^,^^17^

From a laboratory stewardship perspective, the present retrospective workflow simulations provide a practical illustration of how alternative ANCA testing approaches may influence laboratory test utilization in high-volume routine laboratory settings. However, these analyses represent hypothetical estimations within the same retrospective dataset rather than formal diagnostic or cost-effectiveness analyses. Therefore, the findings should not be interpreted as supporting a single optimal testing hierarchy applicable to all clinical settings. Instead, they highlight the heterogeneous laboratory characteristics associated with different testing approaches in routine practice.

This study has several limitations. First, the retrospective design and lack of systematic clinical correlation precluded assessment of true clinical diagnostic performance and prevented evaluation of the relationship between serological findings and confirmed AAV diagnoses. Second, detailed clinical indications and longitudinal ­follow-up data were not uniformly available, limiting stratification according to clinical pretest probability. Third, MPO and PR3 reactivity were evaluated qualitatively using an integrated IIF-based assay platform rather than independent quantitative immunoassays, and signal intensity or antibody titer data were not available for analysis. In addition, because IIF pattern interpretation and MPO/PR3 reactivity assessment were performed within the same integrated assay workflow, complete methodological independence and blinded interpretation could not be ensured. The study was also conducted in a single high-volume tertiary care center, which may limit generalizability to other practice settings. Despite these limitations, the large sample size and real-world laboratory setting provide valuable insight into concordance characteristics and workflow patterns associated with routine ANCA testing in contemporary laboratory practice.

Substantial discordance was observed between IIF patterns and MPO/PR3 reactivity in this large real-world laboratory cohort. Different testing approaches were associated with distinct laboratory workflow characteristics and discordance patterns within the study population. The present findings reflect laboratory concordance characteristics rather than true clinical diagnostic performance, and the clinical significance of discordant serological findings remains uncertain in the absence of systematic clinical correlation. Future prospective studies incorporating detailed clinical and longitudinal data are needed to better define the clinical implications of isolated IIF or MPO/PR3 reactivity in contemporary ANCA testing practice.

## Figures and Tables

**Table 1.** Distribution of Requesting Clinical Departments and Antineutrophil Cytoplasmic Antibodies Test Results in the Study Population

Variable	n (%)
Total patients	8976 (100)
Requesting department	
Rheumatology	3428 (38.2)
Neurology	915 (10.2)
Internal Medicine	874 (9.7)
Pulmonology	777 (8.7)
Gastroenterology	647 (7.2)
Allergy	604 (6.7)
Nephrology	443 (4.9)
IIF results	
Negative	8387 (93.4)
P-ANCA	453 (5.1)
C-ANCA	136 (1.5)
Antigen-specific ANCA antibodies	
MPO positive	153 (1.7)
PR3 positive	159 (1.8)
MPO or PR3 positive	307 (3.4)
MPO and PR3 both positive	5 (0.1)

Only the most frequently requesting clinical departments are shown.

ANCA, anti-neutrophil cytoplasmic antibody; C, cytoplasmic; IIF, indirect immunofluorescence; MPO, myeloperoxidase; P, perinuclear; PR3, proteinase 3.

**Table 2.** Distribution of Indirect Immunofluorescence Patterns According to Myeloperoxidase and Proteinase 3 Antibody Reactivity and Agreement Statistics (Cohen’s Kappa)

IIF pattern	MPO+ /PR3+	MPO+ /PR3−	MPO− /PR3+	MPO− /PR3−	Total	κ (95% CI)
C-ANCA	2	12	64	58	136	0.438 (0.353-0.524)
P-ANCA	3	61	9	380	453	0.191 (0.120-0.261)
IIF negative	0	75	81	8231	8387	

Cohen’s kappa (κ) reflects agreement between IIF pattern and MPO/PR3 antibody positivity: overall (all IIF results vs. any antibody positivity), C-ANCA vs. PR3 positivity, and P-ANCA vs. MPO positivity. κ not applicable for IIF-negative reference category.

ANCA, antineutrophil cytoplasmic antibodies; C, cytoplasmic; IIF, indirect immunofluorescence; MPO, myeloperoxidase; P, perinuclear; PR3, proteinase 3.

## Data Availability

The data that support the findings of this study are available on request from the corresponding author.
